# Chikungunya: an emerging rheumatological pandemic?

**DOI:** 10.46439/rheumatology.2.012

**Published:** 2021

**Authors:** Joshua B. Bilsborrow, J. Kennedy Amaral, Robert Schoen

**Affiliations:** 1 Section of Rheumatology, Allergy and Immunology, Department of Internal Medicine, Yale University School of Medicine, New Haven, Connecticut, United States; 2 Veterans Affairs Connecticut Healthcare System, West Haven VA Medical Center, West Haven, Connecticut, United States; 3 Department of Infectious Diseases and Tropical Medicine, Federal University of Minas Gerais, Belo Horizonte, Minas Gerais, Brazil

**Keywords:** Chikungunya, Alphavirus, Epidemic, Pandemic, Arthritis, Vaccination, COVID-19

## Abstract

Chikungunya virus (CHIKV) has recently emerged alongside Ebolavirus, Zika virus, and SARS-CoV-2 as the causative pathogen for a global pandemic. CHIKV is a single-stranded RNA alphavirus that is transmitted by *Aedes* species mosquitos and has spread beyond its endemic regions in East Africa and South Asia through the Indian Ocean islands, into Southern Europe, and through the Caribbean and the wider Americas. Acute chikungunya fever (CHIKF) is characterized by high fevers, arthralgias, myalgias, headaches, gastrointestinal disturbances, and maculopapular rash. Almost all patients recover from the acute illness, but up to fifty percent develop a chronic rheumatic syndrome with disabling arthritis that can last for months to years. Treatment of acute CHIKF is mainly supportive, while immunomodulation of chronic rheumatic disease with disease modifying anti-rheumatic drugs (DMARDs) such as methotrexate appears to be beneficial. Preventive measures such as mosquito vector control and vaccination could decrease transmission and reduce the burden of chronic disabling post-viral arthritis.

## Introduction

Globalization and the interconnectedness of human societies has increased the potential for regional viral infections to spread across populations with little preexisting immunity. The speed and ease of international travel, increasing urbanization, and climate change also make viral pandemics more likely and more devastating [1]. Thus, it not surprising that the past decade has witnessed multiple international viral epidemics/pandemics, including the 2013–16 West Africa Ebolavirus epidemic [2], the 2015–16 Zika virus epidemic in the Americas [3], and the ongoing SARS-CoV-2 pandemic from December 2019 [4].

The Ebola, Zika, and now SARS-CoV-2 epidemics/pandemics have captured public and scientific attention. Less recognized has been the emergence of the chikungunya virus (CHIKV) pandemic. CHIKV is a single-stranded, positive-sense RNA alphavirus that causes acute chikungunya fever (CHIKF), an illness characterized by high fevers, headaches, arthralgias, myalgias, nausea, vomiting, diarrhea, and crippling fatigue [5]. CHIKV is spread by *Aedes* species mosquitoes and was initially isolated in southern Tanzania in 1952–53. Prior to this discovery, CHIKF had likely been misdiagnosed as dengue for some time given its similar clinical presentation [5]. The name “chikungunya” is derived from the Makonde language and means “that which bends up” or “to become contorted,” reflective of the twisted body posturing affected patients would assume to relieve their severe joint and muscle pains [6].

Chikungunya was endemic in East Africa and South Asia until the first decade of the 21^st^ century [7], whereupon the virus spread globally. Following an indeterminate period of endemicity, CHIKV has emerged as a true global contagion through a series of regional epidemics first through the Indian Ocean region, into southern Europe, and then throughout the Caribbean and the Americas [8–10]. In 2004, following an outbreak in coastal Kenya, CHIKV spread to Réunion and then throughout the Indian Ocean islands [11]. In Europe, a focal outbreak occurred in Italy following the arrival of a traveler from India [9]. In the Americas, the pandemic began in 2013 in the French collectivity of Saint-Martin [12]. The virus reached Brazil in 2014 and has resulted in more than 700,000 confirmed cases over a 4-year period [13]. In the United States, limited endemicity has been established with non-sustained locally acquired cases in Florida and Texas, but infections among returning travelers from endemic regions have been documented in forty-nine states [10].

## Acute Chikungunya Virus Infection

CHIKV is transmitted following a bite from an infected *Aedes* mosquito. Subsequently, after an incubation period averaging two to six days, patients develop fevers, headaches, arthralgias, myalgias, nausea, vomiting, diarrhea, and/or a maculopapular rash. This clinical syndrome has been termed “acute chikungunya fever” (CHIKF) [14]. The joint pain can be severe and is comparable with the “break-bone fever” of acute dengue infection. Arthralgias most often involve the small-to-medium sized joints in a symmetric distribution, notably the metacarpophalangeal (MCP) and inter-phalangeal joints of the hands, the wrists, ankles, and metatarsophalangeal (MTP) joints of the feet. Less often, the medium-to-large joints including the shoulders, elbows, intervertebral joints, hips, and/or knees are affected [15]. Leukopenia (principally lymphopenia) and/or thrombocytopenia can complicate acute febrile infections [11].

Severe disease manifestations are much rarer, but these contribute to greater morbidity and mortality when they occur. Cardiovascular involvement can manifest with myocarditis, dilated cardiomyopathy, and/or vasculopathies [16,17]. Nervous system disease can include meningitis, encephalitis, myelitis, radiculitis, and/or peripheral neuropathies [18]. Ocular manifestations can include keratitis, episcleritis, uveitis, and/or retinitis [19].

CHIKV has a tropism for the epithelium and endothelium, fibroblasts, and macrophages though not for other myeloid-derived lineage cells such as monocytes and dendritic cells [20]. Preferential infection of endothelium-rich vascularized tissues such as the synovium could account for the prominent development of arthralgias and arthritis in patients with acute CHIKF. The inflammatory cytokine profile of acute CHIKF has been characterized by serum elevations of IL-1Ra, IL-1β, IL-6, IL-7, IL-8, IL-12, IL-15, and INF-α in particular [21].

CHIKF usually resolves within ten to fourteen days. The overall reported mortality rate is less than one percent, but can approach ten percent in patients with severe disease manifestations [22]. The mortality rate has also been observed to be higher among neonates, the elderly, and patients with underlying cardiovascular and/or pulmonary conditions [23].

The diagnosis of CHIKF is often made on clinical grounds, especially in resource-limited countries where viral and serological testing may not be available. Most patients are seen in the context of epidemics and live in or have travelled to an endemic region within the prior two weeks. From onset of symptoms, CHIKV viremia lasts five to seven days and can be detected with serum polymerase chain reaction (PCR) testing. Anti-CHIKV IgM antibodies develop around three to eight days into acute infection, waning after one to three months. Anti-CHIKV IgG antibodies develop as acute infection wanes, and these can remain positive for months to years. Anti-CHIKV IgG antibodies are indicative of previous infection [5].

## Chronic Chikungunya Rheumatic Disease

While most patients recover from acute infection, up to fifty percent have been reported to develop a chronic rheumatic syndrome months to years following CHIKF characterized by joint pain, joint swelling, and marked disability [24]. For example, among 152 Colombian patients evaluated at 26 weeks after acute disease onset, persistent arthritis was found in 54%, morning stiffness in 49%, joint edema in 41%, and polyarthralgia and morning stiffness concurrently in 38% [25]. In a Réunion study of 88 patients, chronic arthritis occurred in 93%, 57%, and 47% at 3, 15, and 24 months respectively [26]. In contrast, in another Colombian cohort, persistent arthritis was less frequent, with only 12% of patients affected at 18-month follow-up [24].

Rheumatic features include symmetrical polyarthritis often affecting the hands and feet similar to rheumatoid arthritis, non-specific arthralgias consistent with post-viral arthritis, or asymmetric oligo- or monoarthritis similar to seronegative spondyloarthritis. Post-viral polyarthralgia, fibromyalgia, adhesive capsulitis, and plantar fasciitis can also occur [27]. When these arthritic manifestations last more than 3 months following the onset of CHIKF, the illness can be termed “chronic chikungunya arthritis.” (CCA) [28].

The immunopathogenesis of CCA following acute infection still remains unclear. One main hypothesis has considered CCA as being caused by chronic CHIKV infection within the joints, though evidence for this has remained limited. Hoarau et al. demonstrated the presence of CHIKV proteins and RNA from the synovial macrophages of just one patient with CCA [29]. In larger cohorts of CCA patients from Réunion and Colombia, however, CHIKV RNA could not be identified in synovial fluid nor in synovial tissues through reverse transcriptase PCR testing, mass spectrometry, or viral culturing methods [30,31]. As such, the majority of authors are now postulating CCA as occurring secondary to immune dysregulation following resolution of acute infection. One potential mechanism for this could involve molecular mimicry between host tissue antigens and CHIKV E1 glycoprotein[32], for example, though the specific mechanisms need to be elucidated.

## Prevention of Chikungunya Infection

As chikungunya infection is spread between humans through *Aedes* mosquito vectors, controlling the mosquito populations and/or preventing mosquito bites could retard the spread of the disease. Such vector control methods include draining of open water breeding areas, chemical insecticides, and even potentially genetic engineering of mosquito populations to inhibit viral carriage [33]. Prevention efforts include mosquito netting and personal application of repellants. In regions where public health institutions and resources are limited or lacking, adequate preventive efforts may be difficult to achieve, however.

## Treatment of Acute Chikungunya Fever

Consensus guidelines generally have recommended conservative management for acute chikungunya infection in order to alleviate symptoms such as fevers, headaches, and arthralgias. Analgesics may include acetaminophen/paracetamol, codeine, and/or gabapentinoids. Many authors have recommended against the use of non-steroidal anti-inflammatory drugs (NSAIDs) given the possibility of co-infection with dengue virus, which could potentially worsen coagulopathy. Adjunctive approaches can include physical therapy and temperature-based modalities [34].

Pentosan polysulfate is a semi-synthetic glycosaminoglycan analogue approved for the treatment of interstitial cystitis that is being investigated for its potential efficacy in acute alphavirus infections. In a mouse model of Ross River Virus (RRV) infection, pentosan polysulfate treatment resulted in decreased intra-articular levels of inflammatory cytokines including IL-6, IL-9, CCL2, and G-CSF, and increased levels of the anti-inflammatory cytokine IL-10. Treated animals also had reduced immune cell synovial infiltration and cartilage thinning [35]. Therapeutic efficacy against acute CHIKV infection remains to be determined, however, and there is currently no data for its use in humans.

Fingolimod is a sphingosine-1-phosphate receptor agonist approved for the treatment of multiple sclerosis. In a mouse model of CHIKV infection, fingolimod treatment ameliorated acute joint inflammation, leading to reduced CD4+ T cell synovial infiltration [36]. Data from human studies is not currently available, but this could be another potential therapeutic for acute CHIKF.

Monoclonal antibodies are also being investigated for the treatment of acute CHIKF. SVIR001 is a bioengineered neutralizing human monoclonal antibody that has been shown to decrease CHIKV viral loads in the joint tissues of acutely infected immunocompetent mice. Treatment of CHIKV-infected rhesus macaques resulted in viremia resolution and ameliorated acute disease in regards to joint inflammation, innate immune cell activation, and serum pro-inflammatory cytokine levels [37]. 5F10 and 8B10 are other neutralizing monoclonal antibodies that have shown efficacy as prophylactic and therapeutic treatments for CHIKV-infected AGR129 mice [38]. Moderna, Inc. developed the first antibody therapeutic for use in humans. mRNA-1944 is an intravenous infusion of messenger RNA that leads to the production of the anti-CHIKV monoclonal antibody CHKV-24 in recipients; phase 1 clinical trials have been completed with reports of subjects developing dose-related neutralizing antibody titers within hours of recieving the infusion, with limited adverse clinical effects [39].

## Treatment of Chronic Chikungunya Arthritis

Because the pathogenesis remains uncertain, no consensus exists as to how CCA should be treated. In addition to symptomatic treatment with NSAIDs, a variety of anti-inflammatory therapies, including glucocorticoids, chloroquine, hydroxychloroquine, sulfasalazine, methotrexate, and immune modulating biologic agents including TNF inhibitors, IL-6 receptor antagonists (tocilizumab), and rituximab have been used [27,40]. There is growing consensus for the efficacy and safety of methotrexate, which has the advantage of cost-effectiveness and widespread availability [28], though evidence from randomized controlled trials is still needed.

## Vaccination Research and Development

Mass vaccination of the population in endemic regions, or targeted vaccination of persons at highest risk for severe outcomes or mortality from CHIKF remains a public health goal. There have been numerous anti-chikungunya virus vaccines that have been developed since the 1960s based around various mechanisms, though to date none have entered late-stage clinical trials nor are available to the public [41]. Formalin-inactivated chikungunya vaccines demonstrated stimulation of robust neutralizing antibody titers in both humans and mice [42,43], though other models have been considered more promising for ongoing research and development. Such models include subunit vaccines (based on the CHIKV envelope glycoproteins E1 and/or E2) [44,45], live attenuated vaccines [46–48], recombinant virus vectored vaccines (based upon measles virus [49–51], adenovirus [52,53], or vesicular stomatitis virus) [54], virus-like particle vaccines [55], chimeric vaccines [56], and nucleic acid vaccines [57–60].

In light of the current multi-national efforts to produce a vaccine against SARS-CoV-2, two anti-CHIKV vaccine candidates are worth further mention. López-Camacho and colleagues reported on a viral vectored vaccine combining RNA for CHIKV structural proteins into a replication-deficient chimpanzee adenovirus, ChAdOx1. Preclinical studies showed that immunization of BALB/c mice resulted in robust T cell mediated and humoral immunity against CHIKV, with subsequent human studies planned [53] The Oxford COVID Vaccine Trial Group recently published findings from their phase 1/2 trial of the novel SARS-CoV-2 vaccine, ChAdOx1 nCOV-19, which integrates SARS-CoV-2 membrane spike protein RNA into the same adenovirus vector. Initial results were promising, as the vaccine was generally well tolerated and resulted in robust T cell mediated and humoral immune responses in the subjects [61].

Goyal and colleagues reported on the development of an mRNA vaccine (mRNA-1388) comprising engineered CHIKV mRNA encoding structural polyprotein. This vaccine conferred 100% protection against CHIKV infection in mice, and has progressed through phase 1 human clinical trials to date [60]. This chikungunya vaccine uses the same RNA technology being utilized by Moderna for the COVID-19 vaccine, mRNA-1273, which produces immune responses against the SARS-CoV-2 membrane spike protein. Jackson et al recently published their findings from the phase 1 trial of this SARS-CoV-2 vaccine, which did not show trial-limiting safety concerns and resulted in humoral immune responses in all trial subjects [62].

## Conclusions

Chikungunya has emerged alongside other viral pathogens in causing international epidemics/pandemics during the twenty-first century. CHIKV has spread beyond its historical endemic regions in East Africa and South Asia through immunologically naive populations in the Indian Ocean, Europe, and the Americas. While acute chikungunya infection causes a short-term though painful and disabling febrile illness, its mortality rate is less than one percent among the general population, though is increased among neonates, the elderly, and in those who develop atypical manifestations. Of equal importance is the development of chronic disabling musculoskeletal pain and joint inflammation in a significant proportion of recovered patients, up to 50% in some cohorts though with wide population variability. Absent effective medical treatments or even preventive measures with mosquito vector control and vaccination, chronic chikungunya arthritis has the potential to burden economic and health systems and contribute to poverty and deteriorating quality of life in affected global regions.

## Abbreviations

COVID-19: Coronavirus Disease 2019
SARS-CoV-2: Severe Acute Respiratory Syndrome Coronavirus-2
CHIKV: Chikungunya Virus
RNA: Ribonucleic Acid
CHIKF: Chikungunya Fever
MCP: Metacarpophalangeal
MTP: Metatarsophalangeal
PCR: Polymerase Chain Reaction
IgM: Immunoglobulin M
IgG: Immunoglobulin G
CCA: Chronic Chikungunya Arthritis
NSAIDs: Non-Steroidal Anti-Inflammatory Drugs
RRV: Ross River Virus
IL: Interleukin
CCL: Chemokine Ligand
G-CSF: Granulocyte Colony Stimulating Factor
CD: Cluster of Differentiation
TNF: Tumor Necrosis Factor
BALB/c: Albino immunodeficient laboratory mouse strain
mRNA: messenger Ribonucleic Acid
